# Residual γH2AX foci induced by low dose x-ray radiation in bone marrow mesenchymal stem cells do not cause accelerated senescence in the progeny of irradiated cells

**DOI:** 10.18632/aging.101327

**Published:** 2017-11-21

**Authors:** Margarita Pustovalova, Тatiana A. Astrelina, Anna Grekhova, Natalia Vorobyeva, Anastasia Tsvetkova, Taisia Blokhina, Victoria Nikitina, Yulia Suchkova, Daria Usupzhanova, Vitalyi Brunchukov, Irina Kobzeva, Тatiana Karaseva, Ivan V. Ozerov, Aleksandr Samoylov, Andrey Bushmanov, Sergey Leonov, Evgeny Izumchenko, Alex Zhavoronkov, Dmitry Klokov, Andreyan N. Osipov

**Affiliations:** ^1^ State Research Center - Burnasyan Federal Medical Biophysical Center of Federal Medical Biological Agency, Moscow 123098, Russia; ^2^ Semenov Institute of Chemical Physics, Russian Academy of Sciences, Moscow 119991, Russia; ^3^ Emanuel Institute for Biochemical Physics, Russian Academy of Sciences, Moscow 119991, Russia; ^4^ School of Biological and Medical Physics, Moscow Institute of Physics and Technology, Dolgoprudny, Moscow Region 141700, Russia; ^5^ Insilico Medicine, Inc, ETC, Johns Hopkins University, Baltimore, MD 21218, USA; ^6^ Institute of Cell Biophysics, Russian Academy of Sciences, Pushchino, Moscow Region 142290, Russia; ^7^ Department of Otolaryngology-Head and Neck Cancer Research, Johns Hopkins University, School of Medicine, Baltimore, MD 21218, USA; ^8^ Canadian Nuclear Laboratories, Chalk River, Ontario K0J1P0, Canada; ^9^ University of Ottawa, Ottawa, Ontario, K1N6N5, Canada

**Keywords:** γH2AX foci, cellular senescence, cellular proliferation, low-dose radiation effects, delayed radiation effects

## Abstract

Mechanisms underlying the effects of low-dose ionizing radiation (IR) exposure (10-100 mGy) remain unknown. Here we present a comparative study of early (less than 24h) and delayed (up to 11 post-irradiation passages) radiation effects caused by low (80 mGy) vs intermediate (1000 mGy) dose X-ray exposure in cultured human bone marrow mesenchymal stem cells (MSCs). We show that γН2АХ foci induced by an intermediate dose returned back to the control value by 24 h post-irradiation. In contrast, low-dose irradiation resulted in residual γН2АХ foci still present at 24 h. Notably, these low dose induced residual γН2АХ foci were not co-localized with рАТМ foci and were observed predominantly in the proliferating Кi67 positive (Кi67+) cells. The number of γН2АХ foci and the fraction of nonproliferating (Кi67-) and senescent (SA-β-gal+) cells measured at passage 11 were increased in cultures exposed to an intermediate dose compared to unirradiated controls. These delayed effects were not seen in the progeny of cells that were irradiated with low-dose X-rays, although such exposure resulted in residual γН2АХ foci in directly irradiated cells. Taken together, our results support the hypothesis that the low-dose IR induced residual γH2AХ foci do not play a role in delayed irradiation consequences, associated with cellular senescence in cultured MSCs.

## INTRODUCTION

DNA double-strand breaks (DSBs) are the most crucial DNA lesions introduced by the exposure of cells to ionizing radiation (IR). It is commonly assumed that it is DSBs that trigger the cellular response to IR exposure [1–3]. Approximately 8 out of 10 IR-induced DSBs are repaired by the relatively fast but error-prone non-homologous end-joining (NHEJ) pathway. This can lead to formation of the deleterious microdeletions and chromosome aberrations in the cell [4, 5]. Inaccurately repaired DSBs may lead to cell death, inactivation of the tumor suppressor genes and activation of the oncogenes [6, 7]. Apparently, the accumulation of the microstructural chromosome abnormalities resulting from the incorrect DSB repair plays a major role in driving cellular senescence [8–10].

The major mechanisms underlying the DSB induction and repair are well characterized for high-dose irradiation [11], but not for the low-dose exposures. This stems from the nature of the classical DSB analysis methods, based on the detection of DNA fragmentation (electrophoretic mobility, viscosity, sedimentation, etc.), that did not have sufficient sensitivity to assess changes in the number of DSBs at doses below 10 Gy [12]. To overcome this challenge, highly sensitive methods for DSB analysis have emerged over the last decades. These methods are based on the immunocytochemical detection of various proteins that accumulate at sites of DNA DSBs and are seen as bright dots or so-called DNA repair foci [13–16]. Each DNA repair focus represents a single DNA DSB [17], allowing very accurate quantitative measurements even at low-dose radiation exposures (10-100 mGy) [18]. Histone variant H2AX phosphorylated at DSB sites by several protein kinases (ATM, ATR and DNA-PK) and called γH2AX is the most widely used marker of DNA DSBs [19–23]. A substantial number of reports have been published that examine various aspects of the dynamics of γН2АХ foci formation in mammalian cells exposed to low-dose radiation [24–26], with a focus being the unusually long retention of these foci in low-dose irradiated cells [27–30]. Such long-retained (at 24 h or so post-irradiation) γН2АХ foci are commonly called residual foci and have been associated with a flawed DSB repair process [27] or the overall inducible nature of the DSB repair [28]. They are thought to contribute to genetic instability and therefore are used as an indicator of the risks associated with exposures to low-dose irradiation, including in computer tomography studies [31, 32].

Of particular interest are the studies of the low-dose radiation effects produced in stem cells. Rapidly advancing methods of regenerative medicine and the use of human stem cells, including mesenchymal stem cells (MSCs) [33, 34], in clinical practices are becoming a routine and often require concurrent diagnostic imaging that delivers low doses to patients and cells [35]. High proliferation capacity of stem cells and a potential transmission of the accumulated DNA damage and mutations to the differentiated progeny of exposed cells [36, 37] requires thorough understanding of DNA damage and repair responses in human stem cells. This is particularly important for low-dose diagnostic X-ray procedures. Repair of low-dose induced DSBs has been hypothesized to be inefficient [28], potentially causing various malfunctions in the progeny of the irradiated stem cells, such as accelerated cellular senescence or malignant transformation.

The aim of the present study was to characterize the γН2AX foci formation and dynamics in MSCs after low- (80 mGy) and intermediate- (1000 mGy) dose X-ray exposures. We further sought to study these revealed γН2АХ responses in the context of delayed radiation effects in the progeny of the irradiated MSCs, such as cellular senescence and proliferation capacity.

## RESULTS

### Early radiation effects

#### Low-dose X-rays induces persistent γH2AX foci

We previously reported that the peak of γH2AX foci formation in the human MSCs occured at 0.5 h post-irradiation [18]. The fast phase of the DSB repair culminates by approximately 4-6 h after the exposure, resulting in repair of about 80% DSBs, whereas the slow phase of the DSB repair takes nearly 24 h [38, 39]. Based on these observations, the following post-irradiation time points were chosen for the analysis: 0.5, 4 and 24 h.

**Figure 1:**
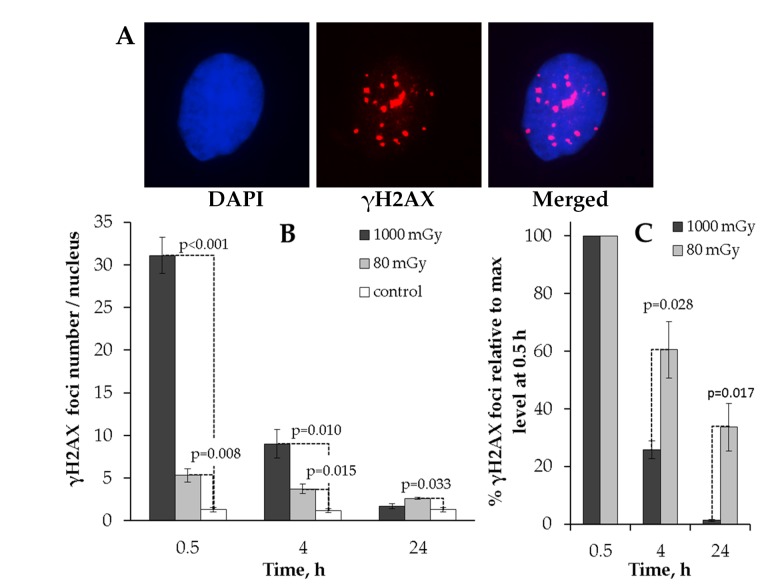
Immunocytochemical analysis of the γH2AX foci in the MSCs irradiated with low (80 mGy) vs. intermediate (1000 mGy) dose of X-ray radiation (**A**) Representative microphotographs of the immunofluorescently stained irradiated MSCs with γH2AX foci showed in red. DAPI nuclear counterstaining is shown in blue. (**B**) Comparative changes in the γH2AX foci number in cells exposed to the low or intermediate dose of X-ray radiation. (**C**) Levels of γН2АХ foci expressed relative to maximum levels at 0.5 h after irradiation demonstrating the difference in the rate of foci clearance after low vs. intermediate doses. Mean values derived from at least three independent experiments are shown. Error bars represent standard error (SE).

The results clearly indicate that the kinetics of γH2AX foci formation after 80 or 1000 mGy irradiation significantly differed from each other (Fig. 1). First, no residual foci were found at 24 h after 1000 mGy irradiation, whereas the number of 80 mGy-induced foci was higher than that seen in the control cells (Fig. 1B). Second, when the number of foci was expressed relative to the peak value at 0.5 h, the two doses produced drastically different kinetics of γН2АХ foci repair. Thus, substantially larger fraction of the initially formed foci disappeared by 4 h in MSCs exposed to 1000 mGy compared to 80 mGy (25.8±3.1% vs. 60.5±9.8% remaining foci, respectively). This gap grew even larger reaching a 10-fold difference at 24 h post-exposure. As seen from Fig 1C, only 1.3±0.4 % of the peak foci number were present in the 1000 mGy irradiated cells and as many as 33.6±8.2 foci were still present in the 80 mGy exposed MSCs.

This data indicates that low-dose X-ray exposure of MSCs can result in residual γН2АХ foci that remain in cells up to 24 h and that this is in contrast to an intermediate dose of X-rays that is followed by efficient elimination of γН2АХ foci by 24 h post-irradiation.

#### Residual γH2AX foci induced by low-dose X-rays are not co-localized with pATM foci

**Figure 2:**
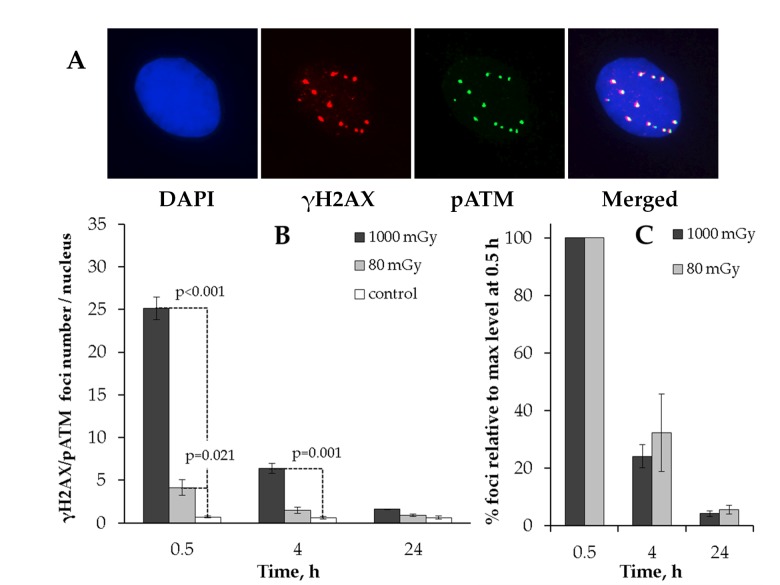
Immunocytochemical analysis of the γH2AX/pAТМ colocalized foci in the MSCs irradiated at low (80 mGy) vs. intermediate (1000 mGy) dose of X-ray radiation (**A**) Representative immunofluorescent images of the irradiated MSCs showing pATM (green) and γH2AX (red) foci. DAPI nuclear counterstaining is shown in blue. (**B**) Quantification of γH2AX/pAТМ colocalized foci in cells exposed to low or intermediate doses of X-ray radiation. (**C**) Levels of pATM-positive γН2АХ (colocalized) foci expressed relative to maximum levels at 0.5 h after irradiation demonstrating the rate of foci clearance after irradiation. Mean values derived from at least three independent experiments are shown. Error bars represent SE.

АТМ protein (ataxia-telangiectasia mutated) is the major kinase that phosphorylates histone Н2AX in response to the IR-induced DNA DSB formation [40], and the slow phase of the IR-induced DSB repair is believed to be ATM-mediated [39]. We next performed a comparative analysis of γH2AX and phosphorylated ATM (pATM) localization in response to either low or intermediate dose of X-rays (Fig. 2).

We first observed that the co-localized foci (γH2AX+/pATM+) resulting from 80 mGy irradiation returned to basal levels by 24 h post-irradiation (Fig. 2B). This result suggested that the residual γН2АХ foci observed after 80 mGy in Fig. 1 may have been pATM-negative. To validate this, we plotted the numbers of γН2АХ+/pATM+ foci relative to maximum levels at 0.5h (Fig. 2C). As can be seen from Fig. 2C, there was no statistically significant difference between the low and intermediate dose groups for γН2АХ+/pATM+ foci.

These results suggest that γН2АХ foci that lack the activated ATM kinase are preferentially selected for long-term retention and thus contribute to the increased residual γH2AX foci rates in the low-dose irradiated MSCs.

#### Persistent γH2AX foci are observed in proliferating but not resting cells

**Figure 3:**
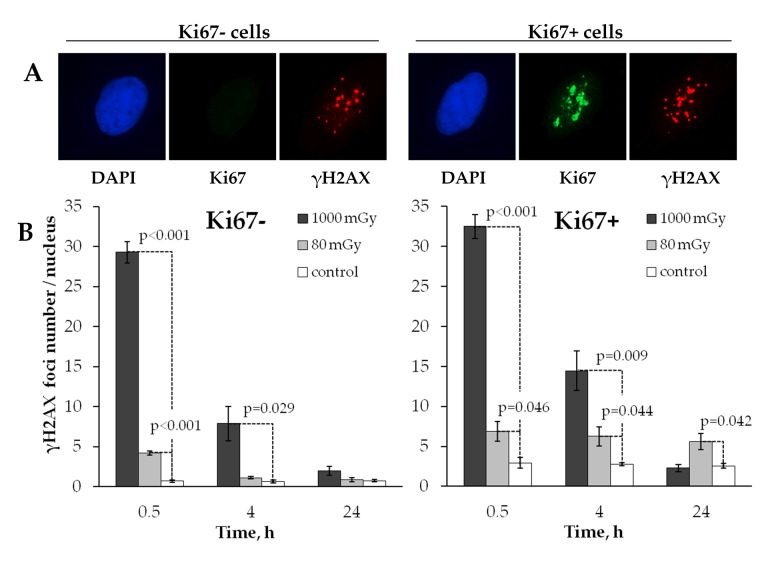
Comparative immunocytochemical analysis of γH2AX foci in resting (Ki67‐) and proliferating (Ki67+) cells (**A**) Representative microphotographs of the immunofluorescently stained irradiated MSCs showing Ki67 (green) and γH2AX foci (red). DAPI nuclear counterstaining is shown in blue. (**B**) Comparative changes in the foci number in resting vs. proliferating cells exposed to the low vs. intermediate dose of X-ray radiation. Mean values derived at least from three independent experiments are shown. Error bars show SE.

The processes of DNA DSB induction and repair are known to differ significantly in proliferating and quiescent cells [41]. Stalled replication forks emerging during the S-phase of the cell cycle are one of the major causes of double-stranded DNA damage in the proliferating cells [42]. Therefore, the mechanisms of residual γН2АX foci persistence may include not only inefficient repair or misrepair of the originally produced DSBs, but also their *de novo* formation in the proliferating cells. To explore this possibility, we next analyzed changes in the radiation induced γН2АX foci formation within quiescent and proliferating cells (Fig. 3A). Since Ki67 expression occurs only in proliferating, but not quiescent cells [43], Ki67 staining was used as a marker of proliferating cells in our work.

Fig. 3B shows that in control unirradiated cells the number of γН2АX foci was approximately 4 times higher (р<0.001) in the proliferating cells compared to the quiescent counterparts (2.74±0.11 vs 0.68±0.11, respectively).

Both the low and the intermediate doses produced similar kinetics of γН2АX foci when observed in quiescent cells in that foci were effectively eliminated by the 24 h time point down to the control level (Fig. 3B, left panel). In proliferating cells, however, a major difference was found between the two treatment groups in how γН2АХ foci behaved after irradiation. While the intermediate dose exposure resulted in the kinetics of γН2АX foci that was similar to one seen in quiescent cells, the foci induced by a low dose did not change over time (Fig. 3B, right panel). This result indicates that residual γH2AX foci produced in human MSCs by low-dose radiation exposure are associated with cellular proliferation activity.

### Delayed radiation effects

#### Low-dose X-rays do not cause an increase in the γH2AX foci number in the progeny of irradiated cells

To evaluate the effect of low-dose irradiation on transgenerational transmission of the DNA lesions or their *de novo* generation in the progeny of irradiated cells, we performed a quantitative analysis of γH2AX foci formation at passages 3, 5, 8 and 11 after IR exposures. Notably, passage 3 after radiation exposure corresponds to passage 6 since the initiation of cell culture of MSCs.

**Figure 4:**
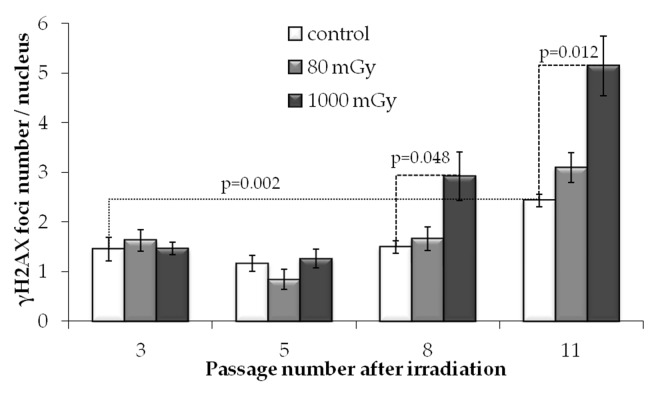
γH2AX foci numbers depending on the passage number in control and irradiated MSCs Mean values derived from at least three independent experiments are shown. Error bars show SE.

In unirradiated cells, the number of γH2AX foci increased almost 2-fold from passage 3 to 11 (р=0.022) (Fig. 4). This observation is consistent with our previous results showing that long-term culture of MSCs leads to accumulation of γH2AX foci [44]. Apparently, the increase in the γH2AX foci number at the late passages of the primary cultures of normal (non-immortalized and non-cancerous) cells may be associated with cellular senescence.

Interestingly and in contrast to the results obtained at 24 h post-irradiation, at passages 3 and 5 we detected no statistically significant differences between the levels of γH2AX foci produced by either 80 or 1000 mGy compared to the non-irradiated control. However, at passages 8 and 11, foci numbers in the progeny of the 1000 mGy irradiated cells exceeded those in the control cultures (Fig. 4). Cultures exposed to 80 mGy were not different from the control cultures over the entire observation period.

These results demonstrate that, in contrast to the inter-mediate-dose irradiation, exposure of MSCs to 80 mGy does not produce persistent increases in the number of γH2AX foci in the progeny of the irradiated cells at passages 3-11 post-irradiation. This was in spite of the fact that 80 mGy-exposed cells demonstrated elevated levels of foci at 24 h after IR treatment.

#### Low-dose X-rays do not decrease proliferation activity in the progeny of irradiated cells

**Figure 5:**
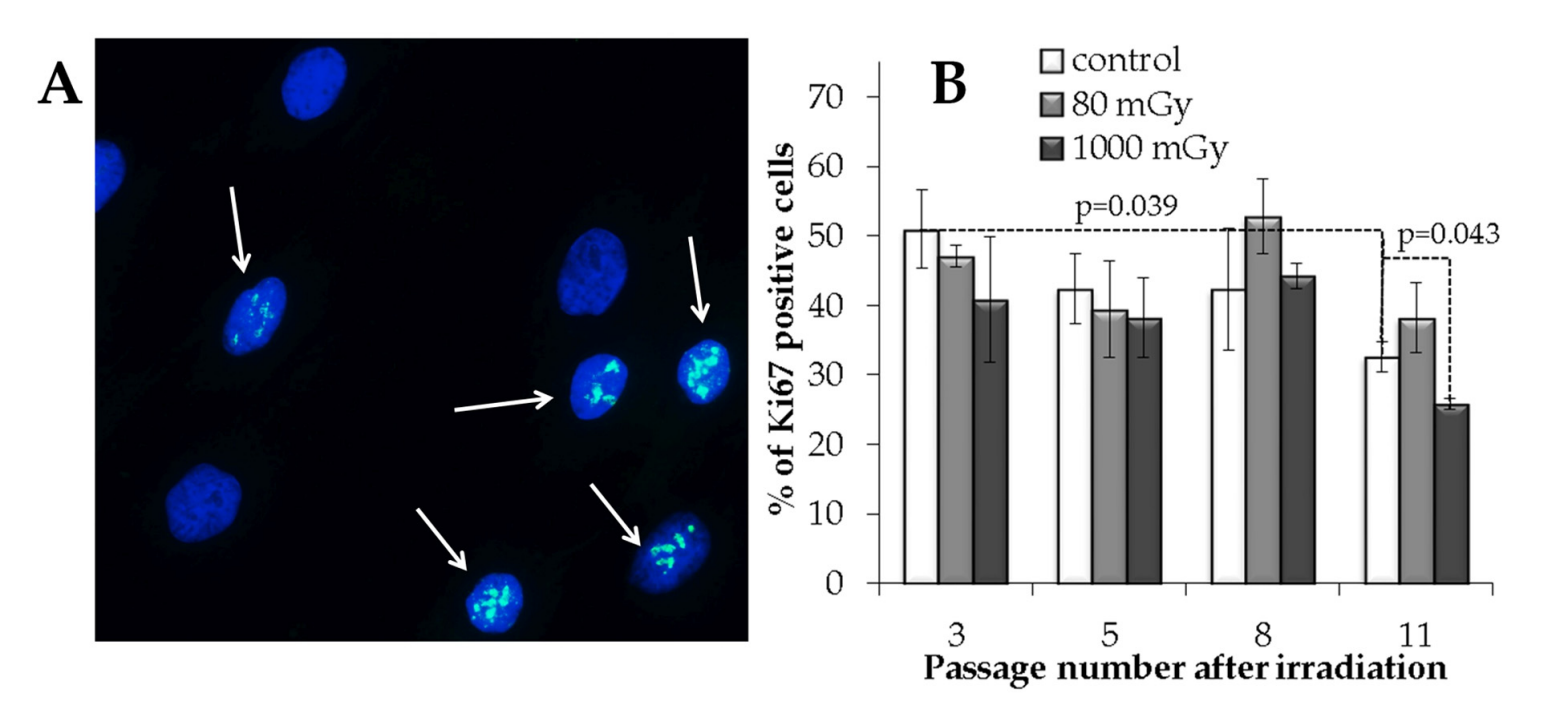
Immunocytochemical analysis of the proliferating (Ki67 positive) cell fraction (**A**) Representative microphotographs of the immunofluorescently labeled MSCs with the Ki67 antibody (Ki67+ cells are marked with the arrows). (**B**) Changes in the percentage of the proliferating cells depending on the passage number in the control and irradiated MSCs. Mean values derived from at least three independent experiments are shown. Error bars show SE.

In order to estimate an overall proliferative capacity, the fraction of Ki67 positive cells was measured in the control and irradiated cell cultures. A statistically significant decrease in the Ki67+ fraction (1.6-fold; p=0.039) was seen from passage 3 to 11 in the control cells (Fig. 5). Cell cultures exposed to an intermediate X-ray dose had a reduced Ki67+ fraction at passage 11 compared to the non-irradiated control cells (р=0.043). On the other hand, the progeny of the low dose irradiated cells did not demonstrate the statistically significant changes in the proliferation capacity compared to the control cell cultures throughout the observation period (Fig. 5).

#### Low-dose X-rays do not accelerate senescence in the progeny of irradiated cells

**Figure 6:**
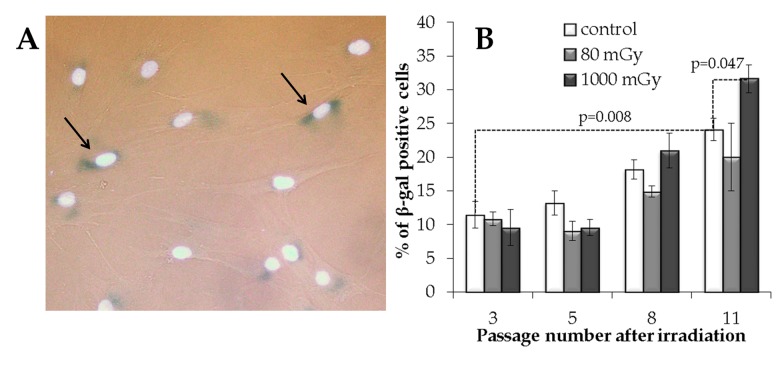
The analysis of the IR exposed SA‐β-gal positive MSCs at various passages (**A**) Representative images of the SA‐β-gal positive cells (marked with arrows - cytoplasm colored in dark blue). Nuclei were counterstained with Hoechst 33342 (bright light blue). (**B**) Changes in the fraction of the SA‐β-gal positive cells depending on the passage number in the control and irradiated MSCs. Mean values derived from at least three independent experiments are shown. Error bars show corresponding SE.

The β-galactosidase (SA-β-gal) staining analysis was performed (Fig. 6A) to evaluate a possible influence of the IR exposure on senescence in the progeny of irradiated cells. This enzyme is commonly used as a marker of cellular senescence since its expression is substantially elevated in senescent cells [45, 46].

The results of the SA-β-gal analysis are shown in Fig. 6. The fraction of the SA-β-gal positive cells gradually increased with passage number in the non-irradiated controls, with an overall 2.1-fold increase from passage 3 to 11 (р=0.008). The accumulation of senescent cells in the progeny of 1000 mGy irradiated MSCs was not different from the control at passages 3 to 8; however, at passage 11, senescence in these cells grew significantly and exceeded that in the control (Fig. 6B). Although the progeny of the low dose irradiated cells demonstrated a trend towards a decrease in the SA-β-gal+ fraction at late passages, this observation was not statistically significant (Fig. 6B).

In summary, our analysis suggests that low-dose X-ray irradiation does not lead to the acceleration of the senescent phenotype formation in the progeny of the human MSCs.

## DISCUSSION

In this study, the early and delayed effects of low- and intermediate-dose X-ray irradiation in cultured human MCSs were investigated. We found that γН2АХ foci produced by a low, but not intermediate, dose were not effectively eliminated, resulting in residual foci still present at 24 h post-exposure. These foci were not associated with ATM-dependent signaling since they were not co-localized with pATM. This is consistent with our previously reported results obtained in human gingival mesenchymal stem cells [18]. We further show that these residual γН2АХ foci were predominantly seen in proliferating cells. Given the observed independence of the low dose associated residual γН2АХ foci of pATM and their preferential appearance in proliferating cells, one may hypothesize that they are not a result of direct radiation-induced DNA damage, but rather are associated with DNA replication processes. Indeed, H2AX can be phosphorylated during replication when errors, such as stalled replication forks, occur and this phosphorylation is ATR, but not ATM, dependent [47, 48]. These errors may be a result of an increased generation of reactive oxygen and nitrogen species characteristic of the S-phase metabo-lism and low-dose radiation can stimulate proliferation [49–51]. Unlike DNA double-strand breaks directly induced by radiation (or by water hydrolysis products upon irradiation), S-phase replication errors typically result in single-stranded DNA lesions and single-ended DNA double-strand breaks that trigger activation of and are processed by the ATR kinase via homologous recombination only [42, 52]. This is a substantially slower DNA repair pathway compared to non-homologous end-joining of double-strand breaks; however, typically about 80 % of the radiation-induced DSBs are processed by the fast NHEJ mechanism [39, 41]. We therefore suggest that in human MSCs low-dose radiation exposure preferentially results in S-phase coupled DNA damage and signaling that activates error-free homologous recombination. This subsequently translates into long-retained pATM-negative γН2АХ foci at 24 h post-irradiation, commonly called residual foci.

Alternative mechanisms may include bystander related processes that are triggered in cells that are not traversed by a photon track [53]. Formation of DSBs in bystander cells has been reported to max between 12 and 48 h [54], consistent with the results presented here. Among various mechanisms suggested for IR-induced responses observed in the bystander cells, the one associated with the mitochondrial dysfunction and reactive oxygen species hypergeneration [55–57] seems to be consistent with our data due to a link to the S-phase metabolism. Indeed, generation of γН2АХ foci in bystander cells was shown to depend on the ATR kinase and occurred predominantly in the S-phase of the cell cycle [58].

Collectively, our early effect results suggest that residual γН2АХ foci observed after low-dose radiation exposure do not originate from the initially produced DNA DSBs that have failed to rejoin. They rather represent secondary *de novo* foci formed in proliferating cells during DNA replication metabolism. Irrespective of their origin, however, one key question is whether these residual γН2АХ foci may present a major concern with respect to long-term consequences of low-dose radiation exposure. We therefore examined various end-points, such as γН2АХ foci numbers, proliferation and senescence, in the progeny of the irradiated cells at passages 3, 5, 8 and 11, corresponding to day 21, 35, 56 and 77 of culture. We show that cells exposed to a low dose did not differ from the control non-irradiated cells at any time-point for all measured end-points. This was in contrast to cells treated with an intermediate X-ray dose that showed changes that can be interpreted as detrimental. Thus, we recorded increasingly higher γН2АХ foci rates, suppression of proliferation and promotion of cellular senescence as the passage number increased in the progeny of 1000 mGy-irradiated cells (Fig. 4, 5, 6).

Long-term consequences of exposure to radiation are associated with health risks. Current uncertainties in measuring the health risks associated with exposures to low-dose radiation present a major scientific problem affecting various areas of human activities, such as radiological protection of the pubic and nuclear industry workers, safety of patients exposed to low-dose diagnostic procedures, space exploration and others. Detrimental health effects of high-dose radiation exposure, mostly measured as increased risk of cancer [59] and used to derive risks after low dose-exposures, have not been unequivocally confirmed experimentally for low doses [60]. However, concerns about the potential of low-dose radiation to cause health detriment, particularly cancer, are frequently raised and discussed [61–63]. Since measuring cancer incidence in experimental animal studies is an extremely difficult task, substantial efforts have been made to find suitable surrogate markers for mutagenicity and carcinogenicity for *in vitro* or *ex vivo* human cell studies. These include dicentric chromosomes [64], micronuclei [65] and γН2АХ foci [66]. However, it is not yet clear whether these are good prediction markers for delayed effects [67]. Physiological systemic changes, such as cellular aging or senescence, may provide a better reference for evaluating a long-term change as a result of low-dose exposure. Indeed, cancer is an aging-related systemic disease and many pathways involved in aging and cancer overlap [68, 69]. Additionally, tissue areas displaying high levels of cellular senescence were shown to eventually become the focal points of tumorigenesis *in vivo*, providing a direct link between senescence and carcinogenesis [70]. Our data showing the lack of long-term consequences, including senescence, after low-dose radiation exposure in human MSCs suggest that residual γН2АХ foci observed at 24 h may not be a good choice for overall evaluation of long-term effects. Indeed, we did not observe increased rates of γН2АХ foci at passages 3 and 5 after irradiation, implying that irradiated MSCs (at least at the doses used here) do not transmit the residual γH2AX foci through the cell divisions, as described by Vaurijoux et al. [71]. At the same time the observed high rates of γН2АХ foci in the progeny (passages 8 and 11) of 1000 mGy-exposed cells is consistent with radiation-induced genome instability [72, 73] or accelerated cellular senescence [74, 75]. Genome instability and senescence are not necessarily linked. Thus, immortalized cancer cell lines do not typically display senescence but their genomes are highly unstable [76, 77]. Therefore, it is possible that in primary cultures of normal human cells senescence, but not genetic instability, is the main contributor to the observed effects. Furthermore, since senescence and γН2АХ foci rates were elevated at late passages in the control cells and thus represent normal aging markers, one may suggest that 1000 mGy irradiation may have intensified the aging-related processes. In contrast, 80 mGy appeared to not affect these. Lastly, given an essential role epigenetic mechanisms play in both long-term responses to radiation exposures [78], including low doses [79, 80], and aging-related alterations [81], our results showing differential long-term responses to low vs. intermediate radiation doses open an exciting new avenue for studying a role of epigenetic mechanisms in the observed phenomenon.

Importantly, the dose of 80 mGy falls within the window of doses delivered to patients during medical diagnostic imaging procedures, such as CT scans [82] that often done in conjunction with stem cell therapy procedures. Such therapies constitute one of the major directions of medicine, called regenerative medicine, that showed great results in treating a variety of severe disorders and that is anticipated to grow exponentially in the near future. Our results therefore provide important knowledge that can be utilized in regenerative medicine with respect to understanding health risks resulting from exposures of human stem cells to low doses of radiation. In particular, our results indicate that although excess γН2АХ foci were present at 24 h post-irradiation with 80 mGy, a finding that is commonly interpreted as presenting mutagenic potential and subsequently health risk, the progeny of the irradiated cells did not display health abnormalities, such as increased senescence, suppressed proliferation and high γН2АХ foci rates. We conclude that accurate inter-pretation of γН2АХ foci measurements may require additional assays, such as quantification of pATM foci, proliferation and senescence, over extended periods of time. Indeed, irregular kinetics of γН2АХ foci observed in this study was also dependent on dose. Therefore, care must be taken when using γН2АХ in biodosimetry or in accessing individual radiosensitivity since time of sampling after irradiation may yield inconsistent results. Furthermore, the lack of insight into the biological relevance of residual γН2АХ foci may lead to a significant overestimation of dose and/or associated health risks.

## MATERIALS AND METHODS

### Bone marrow mesenchymal stem cells isolation and culturing

MSCs we isolated from bоne marrow aspirates of healthy donor (37 years old, male). Ethics approval for the study was granted by the Russian Healthcare Regulation Authority and the Ethics Committee of the Burnasyan Federal Medical Biophysical Center (Reference number: 5A/12.02.2016). Bone marrow aspirates were diluted with phosphate-buffered saline (PBS, Life Technologies, Gibco®, USA) after which mononuclear cells were isolated by Ficoll-Рaque (Lympholyte®, Cedarlane, Canada) density gradient centrifugation (Cedarlane, Canada). Mononuclear cells were then washed with PBS and cultured in MesenCultTM MSC Basal Medium with supplement (StemCellTM Technologies, Canada) and 1% penicillin/streptomycin (Sigma-Aldrich, USA).

The bone marrow MSCs were seeded onto the culture vented flasks with a bottom area of 25 sm^2^. The number of seeded cells was equal 0,3×10^6^ per flask. Cultivation was performed under the standard conditions of CO_2_-incubator (37°С, 5% СО_2_, saturated humidity).

For the immunophenotypic characterization, cells were stained with the panels of antibodies against the following surface markers: CD3, CD13, CD14, CD19, CD25, CD29, CD31, CD34, CD38, CD44, CD45, CD69, CD73, CD90, CD105, CD106, CD166 and HLA-DR (Becton Dickinson, USA). The expression of the surface markers was then analyzed using a BD FACS Canto II (Becton Dickinson Bioscience, USA) flow cytometer. The resulting expression profiles revealed high expression levels (>60% positive cells) for CD90, CD105, CD166, CD44, CD73, medium levels (30-60%) for CD13, CD29 and CD69, and very low levels (<5%) for CD45, CD34, CD133, CD3, CD19, CD25, CD38, CD45, CD106, CD31 markers. This immunophenotype was consistent with the reported immunophenotype for MSCs [83] and did not change in the course of the experiment.

### Irradiation

MSC cells were exposed to the 100 kV X-rays at the dose rate of 40 mGy/min (0.8 mA, 1.5 mm A1 filter) using RUB RUST-M1 X-irradiator (Russia). The cells were irradiated at the 3rd passage from the start of the cultivation in 80 mGy and 1000 mGy doses. Throughout the irradiation time, cells were maintained at 4°C using thermogranules Lab Armour (Life Technologies, USA). The error in the exposure dose was estimated to be within 15%. Cells were returned to the normal growth conditions immediately after the irradiation and maintained for various periods of time before the fixation according to the experiment design.

### Immunoflourescence staining

Cells were fixed on coverslips in 4% paraformaldehyde in PBS (pH 7.4) for 20 min at room temperature followed by two rinses in PBS and permeabilization in 0.3% Triton-X100 (in PBS, pH 7.4) supplemented with 2% bovine serum albumin (BSA) to block non-specific antibody binding. Cells were then incubated for 1 hour at room temperature with primary rabbit monoclonal antibody against γH2AX (dilution 1:200, clone EP854(2)Y, Merck-Millipore, USA) and primary mouse monoclonal antibody against phosphorylated ATM protein (dilution 1:200, clone 10H11.E12, Merck-Millipore, USA) or primary mouse monoclonal antibody against Ki67 protein (dilution 1:400, clone Ki-S5, Merck-Millipore, USA) which were diluted in PBS with 1% BSA. After several rinses with PBS cells were incubated for 1 hour with secondary antibodies IgG (H+L) goat anti-mouse (Alexa Fluor 488 conjugated, dilution 1:600; Merck-Millipore, USA) and goat anti-rabbit (rhodamine conjugated, dilution 1:400; Merck-Millipore, USA) diluted in PBS (pH 7.4) with 1% BSA. Coverslips were then rinsed several times with PBS and mounted on microscope slides with ProLong Gold medium (Life Technologies, USA) with DAPI for DNA counter-staining. Cells were viewed and imaged using Nikon Eclipse Ni-U microscope (Nikon, Japan) equipped with a high definition camera ProgRes MFcool (Jenoptik AG, Germany). Filter sets used were UV-2E/C (340–380 nm excitation and 435–485 nm emission), B-2E/C (465–495 nm excitation and 515–555 nm emission) and Y-2E/C (540–580 nm excitation and 600–660 nm emission). 300-400 cells were imaged for each data point. Foci were counted by manual scoring.

### Analysis of β-galactosidase positive cells

To quantify the proportion of β-galactosidase positive cells the commercial kit «Cellular Senescence Assay» (EMD Millipore, USA, Catalog Number: KAA002) was used. The cells were stained according to supplemented manufacturer protocol with the following modification: at the final РВS washing step, the cell nuclei were stained with 1 μg/ml Hoechst 33342 (Molecular Probes, USA). Such modification significantly improves the quality of counting of β-galactosidase negative cells [46]. The stained cells were visualized using Excitation/Emission Interference Filters (СKX-U: 340-380nm/435-485 nm) on the inverted fluorescent microscope Оlympus СКХ 41 SF (Оlympus, Japan) equipped with Infinity 3-1 (Lumenera Copr., Canada) CCD camera and 20X objective.

### Statistical analysis

Statistical and mathematical analyses of the data were conducted using the Statistica 8.0 software (StatSoft). The results are presented as means of three independent experiments ± standard error. Statistical significance was tested using the Student t-test.
